# How emotional intelligence affects college teachers’ wellbeing in China? The mediating role of work-family support

**DOI:** 10.3389/fpsyg.2025.1517842

**Published:** 2025-04-08

**Authors:** Yongping Li, Lebin Huang, Sobia Naseem, Qichao Shen

**Affiliations:** School of Economics and Management, Hunan University of Arts and Science, Changde, China

**Keywords:** well-being, emotional intelligence, work-family support, college teachers, mediating role

## Abstract

The pursuit of happiness is an individual’s goal in life, and an individual’s fulfillment in life, work, and psychology are essential dimensions of happiness. Emotional intelligence and work-family climate are important factors affecting individual happiness, but the mechanism of their influence on happiness is not well defined. This paper conducted an empirical study on the relationship between college teachers’ emotional intelligence, work-family support and wellbeing through questionnaires and structural equation modeling methods, and the results showed that (1) there are some differences in the demographic characteristics of college teachers’ emotional intelligence, work-family support, and wellbeing; (2) both college teachers’ emotional intelligence and work-family support can positively predict their wellbeing; (3) college teachers’ emotional intelligence positively predicted their work-family support and work-family support partially mediated the relationship between emotional intelligence and wellbeing. The study reveals the internal mechanism by which the emotional intelligence of college teachers affects their wellbeing and that improving their emotional intelligence and work-family support atmosphere can help enhance their wellbeing.

## 1 Introduction

The pursuit of happiness is a fundamental life goal for every college teacher. They dedicate the majority of their time and energy to teaching and nurturing students, from which happiness and a sense of achievement naturally become an important part of their happy lives (Ma and Ma, 2021). The Chinese government is concerned about the wellbeing of teachers as well as working to improve it simultaneously. In early 2018, the Chinese government issued a detailed statement entitled “Opinions on Comprehensively Deepening the Reform of Teacher Development in the New Era (The Central Committee of the Communist Party of China and State Council of the People’s Republic of China, 2018).” This statement aimed to recognize the teachers’ sense of happiness, achievement, and honor in society. It is necessary to care for teachers’ physical and mental health, overcome job burnout and stimulate work enthusiasm. Employee wellbeing is a kind of happiness with specific domain attributes derived from the working environment (Xu et al., 2017). Many scholars define and study employee wellbeing in the context of organizations from the perspectives of subjective wellbeing and psychological wellbeing.

Along with the deepening study of employee wellbeing, the connotation of this topic has been continuously enriched, and the concept of comprehensive wellbeing has emerged. Comprehensive wellbeing is the unity of work, life, and psychological happiness (Zhang et al., 2024). As far as college teachers are concerned, no matter how employee wellbeing is defined, it will be closely related to the emotional competence and work and family atmosphere of college teachers. Emotional competence focuses on the individual’s basic processing and expression of emotions, which is an integral part of emotional intelligence. Emotional intelligence is broader in its fundamentals and conceptualization, which refers to the ability of individuals to monitor their feelings & emotions toward people and situations and use this information to design their thoughts & behavior (Salovey and Mayer, 1990)—the brain processes the emotions for identification, understating and managing the emotions which facilitate emotional thinking. The individual’s job & life satisfaction (Gao et al., 2018; Gao, 2022) and mental health are closely related to emotional intelligence (Luo and Jin, 2016). On the other hand, individuals are subordinate to a specific work-family system, and the work-family atmosphere profoundly influences the perception and experience of employees. Organizational behavior and human resources management are based on the work-family relationships between employees and the organization. In his research, Liu (2019) elucidated the work-family relationships in three phases: work-family conflict, balance, and promotion. Work-family support can be defined as the various domains employees receive, i.e., work domain and family domain during work, which makes their lives happier and more satisfying (Li and Zhao, 2009). Work-family support is not only a form of work-family promotion but also a form of social support, which is closely related to individual job satisfaction, life satisfaction, and mental health (Li and Gao, 2011; Wu et al., 2022; Zeng et al., 2019).

Although existing studies have confirmed that emotional intelligence and work-family support are crucial factors influencing individual wellbeing, they have not fully expounded the underlying mechanisms through which emotional intelligence and work-family support impact employee wellbeing. Moreover, there is a relative rareness of research examining the relationship between emotional intelligence, work-family atmosphere, and employee wellbeing among college teachers. Therefore, this study intends to explore the relationship between emotional intelligence, work-family support, and employee wellbeing among Chinese college teachers, providing a reference framework for enhancing employee wellbeing. Specifically, the objectives of this study are: (1) to measure and evaluate the levels of emotional intelligence, work-family support, and overall employee wellbeing among Chinese college teachers; (2) to examine the correlations between emotional intelligence, work-family support, and employee wellbeing among Chinese college teachers; (3) to uncover the influence mechanisms by which emotional intelligence and work-family support affect the wellbeing of Chinese college teachers. The potential innovations and contributions of this study include: (1) constructing a systematic research framework based on the “employee-work-life” interaction system, incorporating three key variables (emotional intelligence, work-family support, and employee wellbeing) specifically for college teachers; (2) shifting the focus from the negative aspects of work-family relationships and subjective wellbeing to a positive perspective on work-family dynamics and an integrated view of employee wellbeing; (3) revealing the mechanisms by which emotional intelligence and work-family support influence the wellbeing of college teachers, thereby enriching theoretical research on employee wellbeing and offering valuable insights into enhancing the wellbeing of college teachers.

## 2 Theoretical framework and hypothesis

### 2.1 Theoretical framework

#### 2.1.1 Conservation of resources

Hobfoll proposed the Conservation of Resources (COR) theory in 1989, which related to individuals’ ability to conserve, protect, and acquire resources (Hobfoll, 1989). The stress of individuals in this running age is the loss of existing resources and failure to develop new resources (Hobfoll et al., 2018). In 2018, Hobfoll addressed this issue that individuals will use existing resources to generate new resources, which will help to reduce the net loss of resources and actively build & maintain their current resources. The reserve resources help to cope with possible future losses in stressful situations. The resources are considered the theoretical terminology which indicates the things that an individual perceives to help him achieve his goals. Regarding the universal path of individual evaluation on resource value, the healthy body and mind, happy family, happy life and work achievements cherished by all humankind can be regarded as resources. However, regarding the specific path of identifying resource value, high intelligence quotient, high emotional intelligence, self-efficacy, positive psychology, and extensive social support can also be regarded as resources. The latest research on COR theory believes that different resources do not exist independently but interact with each other. According to this theory, employees tend to extensively mobilize individual characteristic resources (such as emotional intelligence, positive psychology, etc.) to obtain a variety of social supports (such as organizational support, family support, etc.) in their work and life and, ultimately achieve the purpose of preserving, protecting and obtaining common cherished resources (such as sense of accomplishment, happiness, etc.).

#### 2.1.2 Job demands-resources model

Demerouti et al. (2001) proposed the Job Demand-Resources Model (JD-R), and this theory is divided into two parts, i.e., job characteristics and job requirements and resources. The determinants that negatively affect job requirements are role conflict, time pressure, job insecurity, etc., which negatively consume the individuals’ energy. From the perspective of individual employees, job requirements are a negative factor, while job resources are positive. The job resources include social support, job autonomy, performance feedback, etc. The job resources promote achieving work goals, personal growth, societal development, job requirements, and related to psychological & physiological costs. Employees’ work is influenced via two paths, which are negative and positive. One is a negative path, which refers to employee job burnout triggered by excessive job requirements and lack of job resources. The other positive path refers to the high work engagement of employees caused by abundant work resources. Job burnout and job engagement are important aspects of job wellbeing. The research of Bauer et al. (2014) also supported the prediction of job characteristics (job demands and job resources) in the JD-R model on employees’ job wellbeing (job burnout and job engagement). According to this theory, employees’ emotional intelligence and work-family support can enrich work resources, stimulate work motivation, and improve work engagement, positively impacting and promoting their job wellbeing.

In summary, Conservation of Resources (COR) theory effectively elucidates an individual’s motivation to leverage one resource domain to acquire or preserve another. Based on the Job Demands-Resources (JD-R) theory, it is speculated that individuals with adequate resources can achieve higher levels of job involvement, job satisfaction, and overall wellbeing. By integrating these two theories, this study aims to construct a research framework that posits college teachers tend to utilize their emotional intelligence to obtain greater work-family support, thereby enhancing their wellbeing. Prior studies have attempted to apply COR and JD-R theories to build research frameworks. For instance, Li et al. (2015) developed a framework to examine the impact of work on happiness, and Bakker et al. (2023) developed a framework about the effect of chronic burnout on dealing with weekly job demands.

### 2.2 Hypothesis

There are two philosophical views on happiness: one is the hedonistic view, and the other is the self-actualization view. Scholars, based on a hedonistic view, consider happiness a subjective experience. However, scholars based on the view of self-actualization believe that happiness is a kind of psychological self-satisfaction. Employee wellbeing is derived from happiness in the work situation and is a kind of happiness with specific domain attributes. Most scholars choose subjective and psychological wellbeing to define and study employee wellbeing. Subjective wellbeing is based on the perspective of hedonism, while psychological wellbeing is based on the perspective of self-actualization (Zhang et al., 2012). Along with the deepening of research, scholars have realized that it is challenging to fully comprehend happiness from a single perspective, so they try to integrate the above two perspectives (Sun et al., 2016). In the perspective of comprehensive wellbeing, employee wellbeing is considered an immediate subjective experience, such as emotions and perceptions in the workplace—lasting objective evaluation of self-actualization and satisfaction experienced by individuals during their work. Employee wellbeing is not only the satisfaction perception of employees at work and in life but also the emotional and psychological experience and satisfaction state of employees at both work and non-work levels. Employee wellbeing mainly includes three structural dimensions: life, work, and psychological wellbeing (Zheng et al., 2015).

Life wellbeing refers to the cognition and perception of employees’ satisfaction with their dimension of life. Work wellbeing refers to the cognition and perception of employees’ satisfaction on the dimension of work. Psychological wellbeing refers to the psychological experience and satisfaction state of employees’ emotions expressed at work and non-work levels. The applied research on employee wellbeing shows that the antecedent variables mainly relate to personality traits, organizational environment, job characteristics, leadership behaviors, work-family interface, interpersonal relationships and social support in the workplace (Zhang et al., 2024).

#### 2.2.1 Emotional intelligence and wellbeing

American psychologist Goleman (1995) indicated that the key to success lies in “emotional intelligence” rather than “intelligence quotient,” which stimulated the attention and enthusiasm of the general public and experts on emotional intelligence. There are two schools of theory about the definition of emotional intelligence. The first is the capability model, which defines emotional intelligence as a kind of capability, including the capability to perceive, understand and manage emotions and the capability to use emotions to promote thinking. The other is the hybrid model, which considers emotional intelligence as a composite structure of capability and trait, mainly including personality traits, emotions, motivation, self-cognitive ability, etc. As far as the structural dimension of emotional intelligence is concerned, scholars generally divide it into four parallel dimensions based on the capability model: self-emotion evaluation capability, others’ emotion evaluation capability, self-emotion regulation capability and emotion application capability (Law et al., 2004; Peciuliauskiene, 2021). Most scholars have studied emotional intelligence as a whole, while recently, some scholars have begun to pay attention to the relationship between various dimensions of emotional intelligence. For example, the study by Lu et al. (2021) considered that the four dimensions of emotional intelligence have a sequence order of effects on specific situations, and the regulation of emotions plays a mediating role in the relationship between the two kinds of evaluation of emotions and learning failure.

Emotional intelligence is an essential factor affecting the wellbeing of college teachers. It can work on the employee-work-family interaction system in two ways, i.e., one is to evaluate and adjust their emotions for a positive experience, and the other is to assess and utilize the feelings of others to improve the atmosphere of the system. Some researches demonstrate that emotional intelligence can positively predict individuals’ subjective wellbeing, while emotional labor (Wang et al., 2018) and self-esteem (Li, 2020) play a partial mediating role between them. Emotional intelligence can also negatively predict individual job burnout and emotional labor partially mediates between these two parts (Yang and Liu, 2024). Emotional intelligence has also been proven to be related to individual mental health (Hong et al., 2021), such as emotional experience (Liang and Wang, 2018), emotional exhaustion (Deng and Sun, 2021), life satisfaction (Yang et al., 2024), mental illness (Nelis et al., 2011), etc. In recent years, more and more scholars have applied emotional intelligence theory to research on teachers. Research shows that higher emotional intelligence can help teachers improve their job satisfaction, teaching efficacy, and mental health (Li et al., 2019). Therefore, this paper proposes the following research hypotheses:

*H_1a_*: College teachers’ emotional intelligence has a significant positive effect on their life wellbeing.

*H_1b_*: College teachers’ emotional intelligence has a significant positive effect on their work wellbeing.

*H_1c_*: College teachers’ emotional intelligence has a significant positive effect on their psychological wellbeing.

#### 2.2.2 Work-family support and wellbeing

Work and family are two crucial components of the social structure of individuals. An excellent work-family relationship is an important demand for employees (Brosch and Binnewies, 2018). Although the research on work-family relationship, which mainly focuses on work-family conflict and work-family balance, began in the 1970s, the positive work-family relationship, such as work-family enhancement, work-family promotion and work-family enrichment, was proposed by scholars only in the last decade (Tao et al., 2019). Work-family support is both a positive work-family relationship and a specific form of social support. French and Shockley (2020) divide the social support individuals receive at work and home into formal support, which refers to policies that support employees in the form of time, services or money, and informal support, which refers to psychological or material resources provided through social relationships. Li and Zhao (2009) developed a work-family support scale based on the background of Chinese culture and divided it into four structural dimensions. The four dimensions are organizational support (the organization provides particular policy support for employees, and the organization makes decisions from the perspective of employees), leadership support (the leader provides specific non-policy support and spiritual support for employees), emotional support (family members offer spiritual and emotional support for employees), and instrumental support (family members provide particular life support for employees). From a specific connotation perspective, organizational support and leadership support primarily pertain to support provided in the work domain (work-to-family support, WSF). Emotional support and instrumental support predominantly relate to support offered in the family domain (family-to-work support, FSW) (Li, 2011a). These four dimensions can also be consolidated into two broader categories.

The support individuals receive from work and family can reduce work-family conflict, maintain work-family balance, and promote work-family gains. Organizational, leadership, and colleague support from the work domain positively correlate with employee happiness (Su et al., 2018). Family-supportive supervisor behavior can promote work prosperity by promoting work-family enrichment (Qin et al., 2022). Organizational and leadership support can also increase employee life satisfaction by promoting work-family enrichment (Jiang et al., 2016). Furthermore, leadership support can also improve employees’ psychological wellbeing by increasing job satisfaction (Swanzy, 2020). Emotional and instrumental support from the family domain directly or indirectly affects an individual’s wellbeing. Spousal support protects mentally stressed employees from emotional exhaustion while alleviating the indirect effects of work tasks on work-family conflict through emotional exhaustion (Pluut et al., 2018). The emotional support of family members can enhance the individual’s sense of work exuberance to obtain higher job satisfaction (He et al., 2023). Recently, some scholars have applied work-family support to research teachers. These researchers believe that support from work and family can help teachers improve their competence (Wang and Liu, 2018) and alleviate work-family conflict (Li, 2011b), thus enhancing their happiness. Hence, the following research hypotheses are proposed in this paper:

*H_2a_*: The work-family support of college teachers significantly positively affects their life wellbeing.

*H_2b_*: The work-family support of college teachers significantly positively affects their work wellbeing.

*H_2c_*: The work-family support of college teachers significantly positively affects their psychological wellbeing.

#### 2.2.3 Emotional intelligence and work-family support

Emotional intelligence is an important ability for individuals to process information based on emotion. Individuals with higher emotional intelligence can better evaluate and deeply understand their own and others’ emotions. They more effectively regulate and utilize their own and others’ emotions to promote successful and efficient interpersonal interactions (Li et al., 2016). They put themselves in a positive and proactive condition and maintain harmonious interpersonal relationships with others. Good interpersonal relationships will help employees improve their work-family environment, create an excellent work-family atmosphere, and get more support and resources from the work and family domain. This is also the concrete manifestation of employees seeking social support and resources using individual characteristic resources. These support and resources will all contribute to an individual’s work and life satisfaction. Therefore, this paper proposes the following research hypotheses:

*H_3_*: College teachers’ emotional intelligence significantly positively influences their work-family support.

*H_4_*: Work-family support partially mediates emotional intelligence’s effect on college teachers’ wellbeing.

### 2.3 Research framework

**Figure 1:**
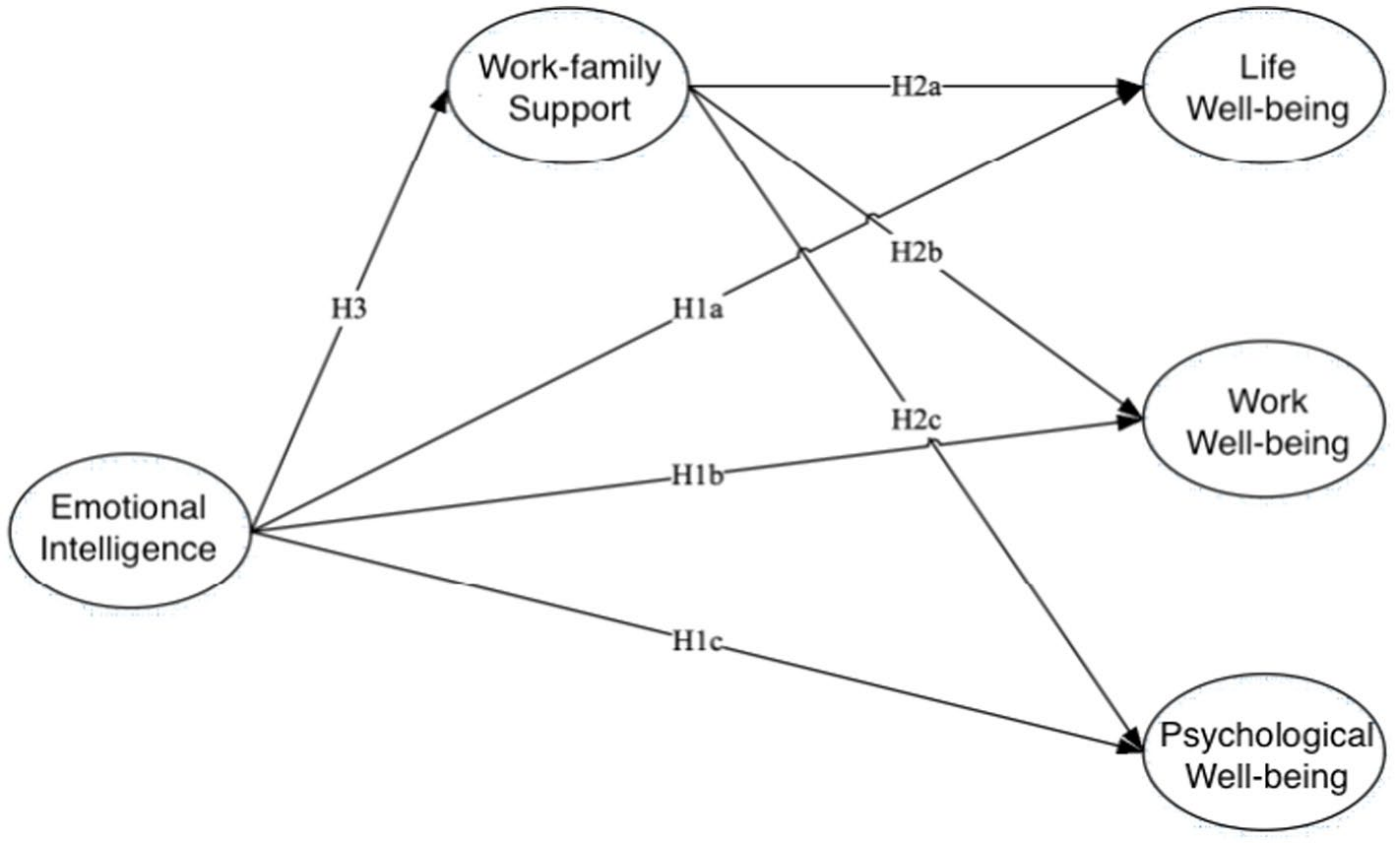
The framework of research on the influence mechanism of emotional intelligence on the wellbeing of college teachers.

This research amalgamated the conservation of resources and job demands-resources model theory with a research hypothesis about the relationship between emotional intelligence, work-family support, and employee wellbeing in college teachers. This paper constructs a research framework on the emotional intelligence mechanism influencing college teachers’ wellbeing in Figure 1.

## 3 Methodology

### 3.1 Research object

The survey was conducted using an anonymous online questionnaire on the widely utilized Wenjuanxing platform, which is popular among Chinese Colleges. It took place between December 29, 2023 and March 6, 2024, with a total of 364 participants consisting of college and university educators who completed the questionnaire. To ensure data authenticity, responses that were completed in less than 1 min were excluded from the analysis. Consequently, we obtained a total of 336 valid samples with an effective rate of 92.3%. The distribution of demographic characteristics within our sample is presented as follows:

(1) Age: 0.6% of the participants were under the age of 25, while 48.2% were between the ages of 25 and 35, 39.3% fell within the range of 35–45, and finally, 11.9% were aged above 45 years old. (2) Gender: The male proportion accounted for approximately 52.1%, whereas females constituted around 47.9%. (3) Marital status: Unmarried individuals comprised 8.6%, married individuals accounted for 90.8%, and divorced individuals represented 0.6%. (4) Education: Individuals with a bachelor’s degree or below constituted 17.6%, those with a master’s degree made up 50.6%, and those with a doctoral degree comprised 31.8%. (5) Health status: Approximately 72.3% reported their health as “good,” 27.0% described it as “average,” while only 0.3% considered it “poor.” (6) School category: Universities classified as either “985” or “211” accounted for 23.5%; non-“985” or “211” universities represented 39.3%; non-“985” or “211” colleges made up 37.2%. (7) Professional title grades: Professors accounted for 10.1%, associate professors represented 17.9%, middle-level titles constituted 48.8%, and primary titles comprised 23.2%. (8) Family income: Those earning over 300,000 yuan amounted to 23.8%; incomes ranging from 80,000 yuan to 300,000 yuan accounted for 74.4%; while only 1.8% had an income below 80,000 yuan. (9) The distribution of working locations is as follows: 25.9% in major cities like Shenzhen, Guangzhou, Shanghai, and Beijing; 36.3% in cities at the provincial level; 33.9% in cities at the prefecture level; and 3.9% in counties or lower administrative divisions. From a demographic perspective, the samples accurately represent the population being studied. The number of valid samples exceeds five times the total number of items on the scale, meeting the requirements for conducting structural equation model analysis.

### 3.2 Research instrument

The questionnaire was divided into primary control and scale measuring factors. The first section (primary control factor) included questions about demographic characteristics, such as gender, age, educational background, marital status, school category, health status, professional title, family income, and work location. The second section consisted of scale items measuring emotional intelligence, work-family support, and employee wellbeing. Initially, the emotional intelligence scale developed by Law et al. (2004) was used in its entirety for this study. It comprised 16 items that assessed self-emotion appraisal (SEA1 to SEA4), regulation of emotion (ROE1 to ROE4), use of emotion (UOE1 to UOE4) and others-emotions appraisal (OEA1 to OEA4). Additionally, the employee wellbeing scale developed by Zheng et al. (2015) was also utilized entirely for this study with a total of 18 items assessing life wellbeing (LWB1 to LWB6), work wellbeing (WWB1 to WWB6) and psychological wellbeing (PWB1 to PWB6). Both intelligence scale and employee wellbeing scale exhibit strong content validity, convergent validity, discriminant validity, and cross-cultural applicability. Finally, the work-family support Scale developed by Li and Zhao (2009) was adapted specifically for university faculty life. It included four dimensions which are organizational support (OS1 to OS5), leadership support (LS1 to LS5), emotional support (ES1 to ES4) and instrumental support (IS1 to IS4). In total there were 18 items on this scale. All three scales were measured using a five-point Likert scale ranging from strongly disagree (= 1) to strongly agree (= 5). Table 1 provides further details on the variables’ structure and item specifics regarding work-family support.

**Table 1:** Variable construction and items of work-family support.

Patent variable	Observed variable	Measurement problem item
Organizational support (OS)	OS1	The college always gives us recognition for our work.
	OS2	The college allows us flexible time to deal with work and family affairs.
	OS3	The college provides a good employment environment and promotion channels and cares about us.
	OS4	The college can meet our material and cultural needs and create a beautiful and comfortable workplace.
	OS5	The college can provide us with good welfare benefits.
Leadership support (LS)	LS1	When my work is affected by family or personal issues, the team leader will understand me.
	LS2	My work performance can always be affirmed and praised by the leader.
	LS3	When my family is in trouble, the leader will give me help.
	LS4	When I have problems with my work, my leaders will take the situation into account and not just criticize.
	LS5	Leaders will be concerned about how the demands of the job affect me personally and at home.
Emotional support (ES)	ES1	When I’m upset about work, my family always understand my feelings
	ES2	When I am tired at work, my family always encourage me.
	ES3	When there is a problem at work, I always talk to my family.
	ES4	When I have a problem at work, my family always comfort me.
Instrumental support (IS)	IS1	My family always give me some personal space after work.
	IS2	When I’m busy at work during a certain time, my family always take on more of the housework.
	IS3	I feel comfortable talking to my family about work.
	IS4	My family are interested in my work.

### 3.3 Research procedure and statistical treatment

Various statistical analysis techniques were utilized in this study. Initially, demographic variables such as emotional intelligence, work-family support, and wellbeing among college teachers were assessed using SPSS 26.0 through means analysis and one-way analysis of variance (ANOVA) on the primary variables. Subsequently, scale reliability, validity, and common method bias were examined for the fully cited Emotional Intelligence Scale and Employee Wellbeing Scale, as well as partial revision of the Work-Family Support Scale. Confirmatory factor analysis (CFA) were selectively conducted using SPSS 26.0 and AMOS 25.0 software tools. Furthermore, a structural equation model (SEM) was constructed to assess how emotional intelligence influences wellbeing among college instructors by performing path analysis and hypothesis testing based on this model. Finally, mediating effects were examined through PROCESS 4.3 plug-in with Bootstrap method using 5,000 repeated samples; a confidence interval of 95% was calculated to ensure result robustness and credibility.

## 4 Results

### 4.1 Statistic description

The means and one-way ANOVA were utilized to analyze the primary variables, and the findings are presented in Table 2. The mean values of the primary variables surpass 3.7 and approach 4.0, indicating a high level of emotional intelligence, work-family support, and employee wellbeing among college teachers. No significant disparities were observed in the mean values of these main variables across gender, family income, and work location factors. However, notable variations were identified in the mean value of emotional intelligence concerning education level, health status, and professional title. Similarly, work-family support exhibited significant differences based on age groups as well as education level and professional title categories. Furthermore, life wellbeing demonstrated noteworthy discrepancies among different age groups, educational backgrounds, and health statuses; work wellbeing displayed distinct distinctions across various age groups along with educational background factors such as health status or professional title; psychological wellbeing showed substantial disparages regarding age groupings as well as marital status categories alongside education level or health status factors. Therefore, it is evident that age groupings along with educational background qualifications have a correlation with the aforementioned main variables.

**Table 2:** Results of mean, standard deviation and one-way ANOVA of emotional intelligence, work-family support and wellbeing of college teachers.

Item	Emotional intelligence			Work-family support			Life wellbeing			Work wellbeing			Psychological wellbeing		
	*F*	*p*	Significance	*F*	*p* value	Significance	*F*	*p*	Significance	*F*	*p*	Significance	*F*	*p*	Significance
Age	1.372	0.079	No	1.405	0.014	Yes	2.196	0.003	Yes	1.978	0.008	Yes	2.661	0.000	Yes
Gender	1.048	0.399	No	1.146	0.190	No	0.680	0.846	No	1.551	0.063	No	0.837	0.656	No
Marital status	0.960	0.541	No	0.906	0.731	No	1.292	0.182	No	1.268	0.199	No	2.652	0.000	Yes
Education	1.838	0.003	Yes	1.391	0.017	Yes	2.035	0.006	Yes	3.105	0.000	Yes	3.137	0.000	Yes
Health status	2.023	0.001	Yes	1.238	0.085	No	1.871	0.014	Yes	1.614	0.048	Yes	2.045	0.008	Yes
School category	1.409	0.063	No	1.036	0.406	No	1.421	0.110	No	1.229	0.228	No	1.227	0.237	No
Professional title grades	1.470	0.043	Yes	1.680	0.000	Yes	1.565	0.059	No	1.901	0.012	Yes	2.400	0.001	Yes
Family income	1.207	0.197	No	1.039	0.399	No	0.334	0.997	No	1.482	0.085	No	0.653	0.856	No
Work location	1.053	0.391	No	0.902	0.739	No	0.692	0.835	No	1.263	0.202	No	1.428	0.116	No
Mean value	3.972			3.894			3.792			3.972			4.029		
Standard deviation	0.524			0.651			0.708			0.664			0.579		

*p < 0.05 means significance.*

### 4.2 Reliability test

The survey used in this research consisted of 11 different subscales, totaling 52 items. The data collected from the survey were analyzed using SPSS 26.0 software, and an overall Cronbach’s alpha coefficient of 0.948 was calculated for the 11 subscales. Except for the self-emotion appraisal (SEA) subscales, which had a Cronbach’s alpha coefficient of 0.771, all other f subscales showed coefficients higher than 0.8. The findings from the data analysis clearly demonstrate that all scales utilized in this study exhibit strong consistency, stability, and reliability.

### 4.3 Validity test

#### 4.3.1 Construct validity

Given that the work-family support scale utilized in this study has been adapted to better reflect the life context of college teachers, we developed two competing full-variable confirmatory factor models and validated these models using AMOS 25.0 software. The fit indices for both models were calculated separately, as detailed in Table 3. Upon comparing the fit indices of the two models, it is evident that the four-factor work-family support model named WFS4 exhibits a marginally superior fit compared to the two-factor work-family support model named WFS2. Consequently, the subsequent data analysis in this study was conducted based on the WFS4.

**Table 3:** Results of full variable confirmatory factor analysis.

Model	Factors	CMIN/DF	RMSEA	CFI	TLI	NFI
WFS4	SEA, ROE, UOE, OEA, OS, LS, ES, IS, LWB, WWB, PWB	1.959	0.053	0.895	0.885	0.808
WFS2	SEA, ROE, UOE, OEA, OS+LS, ES + IS, LWB, WWB, PWB	1.995	0.054	0.889	0.881	0.801

#### 4.3.2 Convergent validity

The confirmatory factor analysis of the collected questionnaire data was conducted using AMOS version 25.0 software, and the results are presented in Table 4. All observed variables in this study serve as indicators of the underlying latent variable. These indicators can reflect their corresponding potential variables. Therefore, these latent variables are reflective. Except for two observed variables, SEA4 and PWB6, the standardized factor loadings were slightly lower than the critical value 0.7, and the standardized factor loadings of other observed variables were all greater than the critical value 0.7. Additionally, the calculated composite reliability (CR) exceeded 0.8, which is well above the critical value of 0.7, while the average variance extracted (AVE) also surpassed 0.5. These findings suggest that the scales utilized in this study possess strong convergent validity.

**Table 4:** Reliability, convergent validity of variables.

Latent variables	Observed variables	Standardized factor loading	No. of items	Cronbach’s alpha	Composite reliability	Average variance extracted
SEA	SEA1	0.723	4	0.810	0.8116	0.5187
	SEA2	0.713				
	SEA3	0.746				
	SEA4	0.698				
ROE	ROE1	0.756	4	0.841	0.8435	0.5741
	ROE2	0.757				
	ROE3	0.733				
	ROE4	0.784				
UOE	UOE1	0.746	4	0.842	0.8422	0.5717
	UOE2	0.763				
	UOE3	0.773				
	UOE4	0.742				
OEA	OEA1	0.735	4	0.850	0.8506	0.5875
	OEA2	0.775				
	OEA3	0.786				
	OEA4	0.769				
OS	OS1	0.806	5	0.876	0.8819	0.5996
	OS2	0.696				
	OS3	0.774				
	OS4	0.812				
	OS5	0.778				
LS	LS1	0.717	5	0.858	0.8582	0.5480
	LS2	0.781				
	LS3	0.724				
	LS4	0.718				
	LS5	0.759				
ES	ES1	0.782	4	0.860	0.8616	0.6093
	ES2	0.781				
	ES3	0.728				
	ES4	0.828				
IS	IS1	0.724	4	0.825	0.8261	0.5429
	IS2	0.738				
	IS3	0.751				
	IS4	0.734				
LWB	LWB1	0.740	6	0.886	0.8875	0.5683
	LWB2	0.777				
	LWB3	0.723				
	LWB4	0.778				
	LWB5	0.717				
	LWB6	0.785				
WWB	WWB1	0.748	6	0.882	0.8838	0.5596
	WWB2	0.814				
	WWB3	0.730				
	WWB4	0.754				
	WWB5	0.730				
	WWB6	0.708				
PWB	PWB1	0.709	6	0.860	0.8606	0.5073
	PWB2	0.707				
	PWB3	0.716				
	PWB4	0.722				
	PWB5	0.723				
	PWB6	0.696				

#### 4.3.3 Discriminate validity

The questionnaire data was analyzed for correlation using SPSS 26.0 software. Pearson correlation coefficients were computed for the latent variables and compared to the square roots of the average variance extracted (AVE). As shown in Table 5, all latent variables displayed higher square roots of AVE than their corresponding Pearson correlation coefficients. These results suggest that the scale utilized in this study exhibits strong discriminant validity.

**Table 5:** Analysis table of discriminate validity.

Variables	AVE	SEA	ROE	UOE	OEA	OS	LS	ES	IS	LWB	WWB	PWB
SEA	0.5187	0.720										
ROE	0.5741	0.485	0.758									
UOE	0.5717	0.500	0.527	0.756								
OEA	0.5875	0.380	0.513	0.437	0.766							
OS	0.5996	0.400	0.562	0.532	0.379	0.774						
LS	0.5480	0.456	0.548	0.525	0.442	0.691	0.740					
ES	0.6093	0.342	0.454	0.559	0.408	0.630	0.650	0.781				
IS	0.5429	0.332	0.488	0.584	0.432	0.626	0.661	0.714	0.737			
LWB	0.5683	0.402	0.582	0.553	0.473	0.709	0.678	0.636	0.692	0.754		
WWB	0.5596	0.427	0.519	0.633	0.417	0.728	0.675	0.650	0.707	0.689	0.748	
PWB	0.5073	0.482	0.552	0.669	0.479	0.667	0.655	0.660	0.677	0.707	0.719	0.712

The diagonal elements represent the square root of AVE.

### 4.4 Common method bias test

Considering that all the data in this study are derived solely from a questionnaire survey conducted during the same period, it is crucial to examine the measurement data of the scale for potential common method bias. To test for common method, bias effectively, we constructed two confirmatory factor analysis models: model_1, which includes all variables, and model_2, which incorporates an additional common method factor. We then compared key fit indices between model_1 and model_2 (as presented in Table 6): ΔRMSEA = 0.004, ΔSRMR = 0.007; both these change values are below the critical threshold of 0.05. Furthermore, ΔCFI = 0.020, ΔTLI = 0.018, ΔNFI = 0.022; none of these three change values exceed the critical threshold of 0.1 either. These results indicate that there is no significant improvement observed in the confirmatory factor model after incorporating the common method factor; thus, suggesting that there is no substantial evidence supporting the presence of common method bias within our measured data—thereby confirming successful completion of our common method bias test.

**Table 6:** Comparison of leading fitting indicators of Model 1 and Model 2.

Model	CMIN/DF	GFI	RMSEA	SRMR	RMR	CFI	TLI	NFI
model_1	1.959	0.802	0.053	0.045	0.033	0.895	0.885	0.808
model_2	1.810	0.806	0.049	0.038	0.027	0.915	0.903	0.830
△	−0.149	0.004	−0.004	−0.007	−0.006	0.020	0.018	0.022

### 4.5 Fitting of a model

The constructed model underwent validation using AMOS 25.0 software, with the structural equation model’s fit index, CMIN/DF, calculated to be 2.946. This value is below the recommended threshold of 3 and significantly lower than the critical value of 5. Furthermore, RMR was determined to be 0.031, which falls below the critical value of 0.05; RMSEA measured at 0.076, below the recommended threshold of 0.08 and far less than the critical value of 1.0; GFI scored at a level of 0.824 while NFI reached a value of 0.857; RFI stood at a level of 0.841 and TLI achieved a score of 0.889—all exceeding the critical threshold set at or above 0.800; CFI obtained a score of 0.900 while IFI reached 0.901—both meeting or surpassing the recommended threshold set at 0.900 or higher. Different fit indices are employed to assess the adequacy of structural equation models, encompassing CMIN/DF, RMR, RMSEA, NFI, RFI, IFI, TLI, CFI, AGFI, and GFI. It is widely acknowledged that when a model satisfies multiple indices concurrently, it indicates a high level of fitness. Thus, it can be concluded that there is good alignment between theoretical model constructed in this study and data with acceptable levels for both model fit and parsimony.

### 4.6 Hypothesis testing

**Figure 2:**
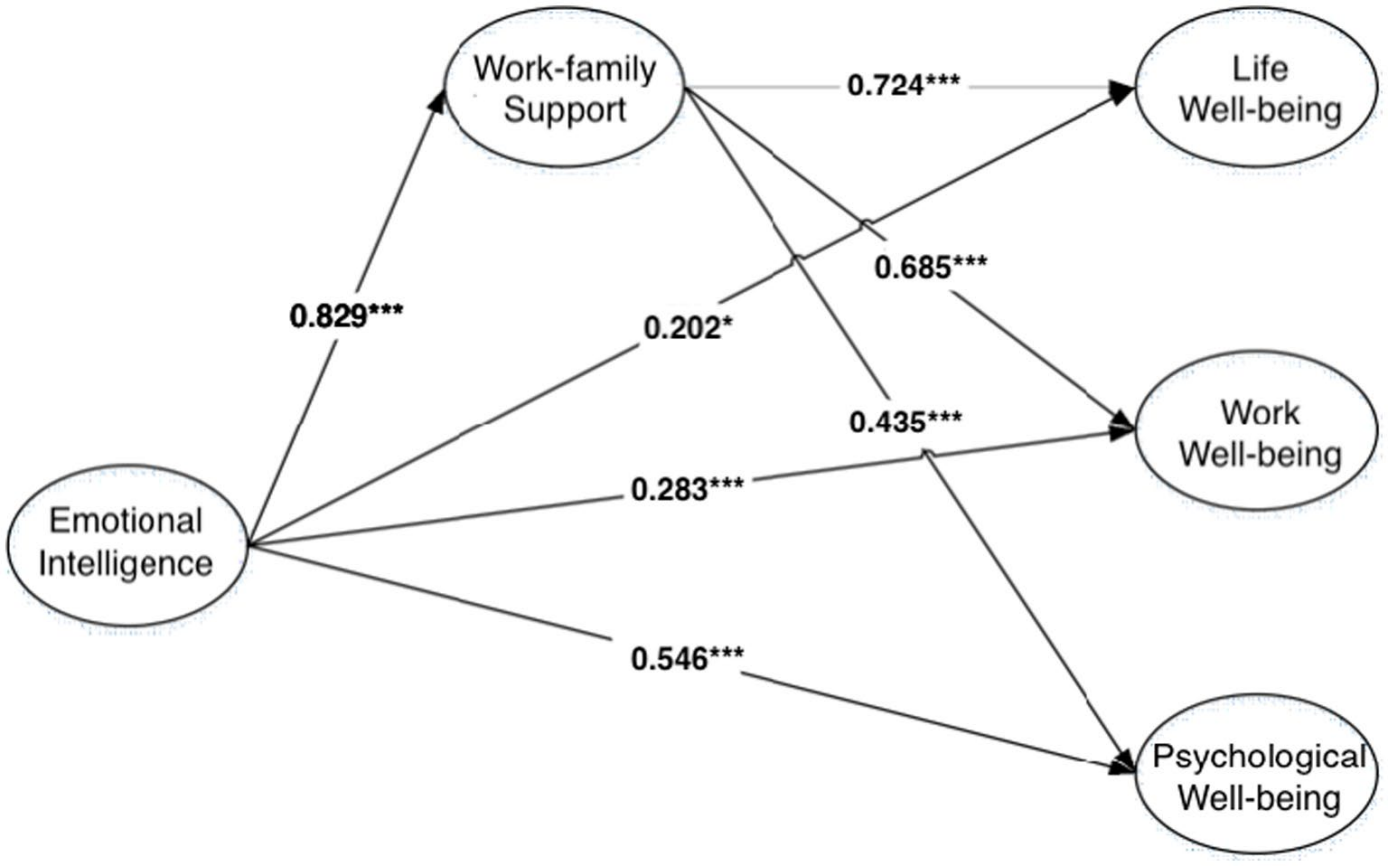
Analysis result of the impact of emotional intelligence on the employee wellbeing of college teachers.

In this study, the constructed model was analyzed using AMOS 25.0 software to examine the relationships and influence coefficients among the latent variables, as shown in Figure 2. The results of hypothesis testing can be found in Table 7. It is evident from Table 8 that all standardized path coefficients demonstrate a positive influence between the latent variable paths mentioned earlier. Furthermore, upon closer examination of their significance, it is observed that all paths exhibit critical ratio (C.R.) values exceeding 1.96 and *p*-values below 0.05, indicating significant influences of these paths and thus supporting research hypotheses H_1a_, H_1b_, H_1c_, H_2a_, H_2b_, H_2c_, and H_3_. The maximum Variance Inflation Factor (VIF) for the independent variables in the regression equations of the path analysis model consistently remains below 5, well below the commonly accepted threshold of 10, thereby indicating the absence of significant multicollinearity concerns.

**Table 7:** Results of hypothesis testing.

Research hypothesis	Path	Standardized path coefficient	Path coefficient	Standard deviation	Critical ratio	Significance	Result
H1a	Emotional intelligence ↓ Life wellbeing	0.202	0.324	0.144	2.250	*	Support
H1b	Emotional intelligence ↓ Work wellbeing	0.283	0.408	0.124	3.290	***	Support
H1c	Emotional intelligence ↓ Psychological wellbeing	0.546	0.640	0.124	5.161	***	Support
H2a	Work-family support ↓ Life wellbeing	0.724	0.729	0.095	7.674	***	Support
H2b	Work-family support ↓ Work wellbeing	0.685	0.620	0.082	7.561	***	Support
H2c	Work-family support ↓ Psychological wellbeing	0.435	0.319	0.068	4.691	***	Support
H3	Emotional intelligence ↓ Work-family support	0.829	1.323	0.134	9.873	***	Support

The symbols *, **, and *** indicate statistical significance at the levels of 0.05, 0.01, and 0.001 correspondingly.

**Table 8:** The mediating role of work-family support in the relationship between emotional intelligence and life wellbeing.

Path	Total effect	Direct effect	Mediating effects	*t* value	95%LLCI	95%ULCI
Emotional intelligence X → Life wellbeing Y (c)	0.6509			15.6678^***^	0.5691	0.7326
Emotional intelligence X → Work-family support M (a)		0.6839		17.1298^***^	0.6053	0.7624
Work-family support M → Life wellbeing Y (b)		0.6176		13.4589^***^	0.5274	0.7079
Emotional intelligence X → Life wellbeing Y (c′)		0.2285		4.9789^***^	0.1382	0.3188
Emotional intelligence X → Work-family support M → Life wellbeing Y (ab)			0.4224		0.3212	0.5381
Effect size (ab/c*100%)	64.8948					

**Table 9:** The mediating role of work-family support in the relationship between emotional intelligence and work wellbeing.

Path	Total effect	Direct effect	Mediating effects	*t* value	95%LLCI	95%ULCI
Emotional intelligence X → Work wellbeing Y (c)	0.6415			15.2809^***^	0.5589	0.7240
Emotional intelligence X → Work-family support M (a)		0.6839		17.1298^***^	0.6053	0.7624
Work-family support M → Work wellbeing Y (b)		0.1941		4.3020^***^	0.1053	0.2828
Emotional intelligence X → Work wellbeing Y (c′)		0.6542		14.5028^***^	0.5655	0.7429
Emotional intelligence X → Work-family support M → Work wellbeing (ab)			0.4474		0.3162	0.5974
Effect size (ab/c*100%)	69.7428					

### 4.7 Mediation test

To investigate the potential role of work-family support in mediating the connection between emotional intelligence and life wellbeing, work wellbeing and psychological wellbeing among college teachers, this study utilized Model 4 (mediating model) from PROCESS 4.3 plug-in. The Bootstrap method was employed to conduct 5,000 iterations to test the mediation effect within the model. Subsequently, significance tests were conducted on regression coefficients c, a, b, ab, and c′, along with calculation of their respective 95% confidence intervals and effect sizes for mediation. The results pertaining to the mediating effect have been presented in Tables 8–10 as per the analysis conducted.

**Table 10:** The mediating role of work-family support in the relationship between emotional intelligence and psychological wellbeing.

Path	Total effect	Direct effect	Mediating effects	*t* value	95%LLCI	95%ULCI
Emotional intelligence X → Psychological wellbeing Y (c)	0.7007			17.9468^***^	0.6239	0.7775
Emotional intelligence X → Work-family support M (a)		0.6839		17.1298^***^	0.6053	0.7624
Work-family support M → Psychological wellbeing Y (b)		0.3438		7.5856^***^	0.2546	0.4330
Emotional intelligence X → Psychological wellbeing Y (c′)		0.5218		11.5135^***^	0.4327	0.6110
Emotional intelligence X → Work-family support M → Psychological wellbeing (ab)			0.3569		0.2395	0.5026
Effect size (ab/c*100%)	50.9348					

According to the mediation analysis method, model, and process proposed by Wen and Ye (2014), Firstly, this study demonstrates the significant total benefit coefficient (c) of emotional intelligence among college teachers on their life wellbeing, work wellbeing, and psychological wellbeing. This implies that the hypothesis of mediating variables in the emotional intelligence of college teachers and employee wellbeing can be posited. Then, it reveals that both the effect coefficient (a) of emotional intelligence on work-family support among college teachers and the effect coefficient (b) of work-family support on their life wellbeing, work wellbeing, and psychological wellbeing are statistically significant, indicating a significant indirect effect. Finally, it confirms that the direct effect (c′) of emotional intelligence among college teachers on their life wellbeing, work wellbeing, and psychological wellbeing remains significant. Moreover, ab and c′ share the same sign which suggests that work-family support plays a partial mediating role between emotional intelligence and happiness for college teachers. The mediation effect accounts for approximately 64.89% in terms of life happiness; about 69.74% in terms of job wellbeing; and around 50.93% in terms of psychological wellbeing.

In accordance with the mediation analysis conducted by Fang and Zhang (2013) using the coefficient product ab test, the Bootstrap method was utilized to investigate the mediating effect of college teachers’ work-family support on emotional intelligence and wellbeing. The results revealed that the 95% confidence interval (CI) for the direct effect c′ of emotional intelligence among college teachers on life wellbeing was (0.1382, 0.3188), while the 95% CI for the indirect effect ab ranged from (0.3212, 0.5381). Similarly, the 95% CI for the direct effect c′ of emotional intelligence among college teachers on work wellbeing was found to be (0.5655, 0.7429), with a corresponding indirect effect ab ranging from (0.3162, 0.5974). Furthermore, the study demonstrated that the 95% CI for the direct effect c’ of emotional intelligence among college teachers on psychological wellbeing fell within (0.4327, 0.6110), and its associated indirect effect ab ranged from (0.2395, 0.5026). Importantly, all these aforementioned confidence intervals exclude zero values signifying significant effects in both paths examined and thus supporting research hypothesis H4.

## 5 Cross-validation of qualitative studies

### 5.1 Data collection

We randomly selected 16 college teachers who had participated in the preliminary questionnaire survey for one-on-one in-depth interviews. The participants represented a diverse range of age groups and professional titles, with females comprising 56.25% of the sample. Their perspectives were considered representative of the broader population. The interview outline was developed based on the findings from the previous quantitative study. Interviewees were asked to assess their emotional intelligence, work and family atmosphere, job satisfaction, life satisfaction, and mental health, as well as provide insights into the relationships between these factors. With the consent of the participants, the entire interview process was audio-recorded. On average, each interview lasted 32.4 min.

### 5.2 Data analysis and results

The recorded data were transcribed to yield a total of 10,645 Chinese characters. We employed a three-level abstraction process (open coding, axial coding, and selective coding) for the analysis. First, during the open coding phase, four researchers meticulously reviewed the interview transcripts, conceptualized and categorized the data according to the research topic, and established a unified coding framework to guide subsequent coding and analysis. Second, in the axial coding stage, we refined and integrated the identified concepts into broader categories that reflect emotional intelligence, work-family support (including work-to-family support and family-to-work support), and employee wellbeing (encompassing work wellbeing, life wellbeing, and psychological wellbeing). Finally, in the selective coding phase, we conducted a thorough review of the interview texts, and the logical axis of “conditions-process-outcomes” became increasingly clear, elucidating the mechanism by which emotional intelligence influences the overall wellbeing of college teachers.

The qualitative analysis corroborated the findings of the quantitative research, providing further evidence for the relationship between emotional intelligence, work-family support, and employee wellbeing among college teachers. First, the responses indicated that individuals with higher emotional intelligence exhibited superior performance both at work and at home, and experienced greater levels of happiness. Interviewee 9 remarked, “People around me think I’m emotionally intelligent, and honestly, I agree… I’m really happy with my life and work because they match what I hoped for… My family and colleagues are great—we get along super well. My colleagues appreciate me a lot, and I just got promoted to professor. I feel really good about how I’m doing at work.” Second, the respondents’ feedback confirmed that individuals who received greater work-family support were better equipped to handle challenges in both their professional and personal lives. Consequently, they were more likely to experience higher levels of work and life satisfaction as well as a stronger sense of accomplishment. Interviewee 4 mentioned, “The working environment and atmosphere at the school are excellent, and the salary adequately supports my standard of living… As a teacher, the flexible working hours allow me to balance my professional responsibilities with family commitments… My wife is a stay-at-home mother who manages the household efficiently, enabling me to dedicate sufficient time and energy to teaching and research… I am deeply satisfied with my job, cherish my family, and feel optimistic about the future.” In contrast, Interviewee 12 stated, “I just started here not too long ago. I’m new to the place and a bit introverted, so I haven’t made many friends yet… The teaching and research workload is really intense, keeping me busy all day… My schedule is all over the place, and I end up staying up late a lot. To be honest, my life and work are pretty rough right now. I’ve even thought about quitting.” Furthermore, the responses indicated that individuals with higher emotional intelligence tended to maintain superior relationships and receive greater work-family support. Interviewee 8 mentioned, “I’m really empathetic and I always try to keep a good relationship with both my leaders and colleagues. They seem to like me a lot and are more than happy to lend a hand when I need it. I care deeply about my family, and my husband is super supportive of my work. He even offers to look after the kids.” In contrast, Interviewee 12 stated, “I just started here not too long ago. I’m new to the place and kind of shy, so I haven’t made many friends yet. The teaching and research workload is really intense, keeping me busy all day. My schedule is all over the place, and I end up staying up late a lot. To be honest, things aren’t going great for me right now… I’ve even thought about quitting.” Finally, the responses enhance our understanding of the mediating role of work-family support in the relationship between emotional intelligence and employee wellbeing. As highlighted by Interviewee 15, “To get my leaders and coworkers on my side, I sometimes give them little gifts, and they usually lend me a hand. I’m pretty good at understanding people and knowing what makes them happy, which helps me keep my family supportive of my work. Overall, I really enjoy my job, love my family, and try to stay positive and steady.”

## 6 Discussion and conclusion

This research is developed based on a model grounded in resource conservation and job demand-resource theory to examine the mediating role of work-family support in the relationship between emotional intelligence and wellbeing among Chinese university teachers. The empirical findings of the research support the proposed hypothetical design.

### 6.1 Emotional intelligence and wellbeing

A significant positive relationship between the emotional intelligence of college teachers and their overall wellbeing in life, work, and psychological wellbeing is observed. In other words, higher levels of emotional intelligence among college teachers are associated with greater wellbeing. These findings align with the conclusions of prior studies (Brackett et al., 2010; Salavera et al., 2020). The empirical analysis results indicate that grounded in the emotion regulation mechanism, cognitive evaluation system and emotional intelligence play a crucial role in emotion perception, motivation, and emotion regulation. Research has demonstrated that the average correlation coefficient between emotional intelligence and subjective wellbeing is *r* = 0.32 (Sánchez Álvarez et al., 2016). Individuals with elevated emotional intelligence possess superior abilities in perceiving, evaluating, and expressing their own emotions. They also demonstrate heightened sensitivity toward others’ emotions and excel in understanding, predicting, and utilizing emotional responses from others. Moreover, individuals with high emotional intelligence exhibit enhanced resilience against psychological difficulties while effectively regulating their emotions to foster constructive behavior and individual achievement. Previous research has consistently shown that high emotional intelligence contributes to increased job satisfaction, life contentment (Rogowska and Meres, 2022), and emotional stability. Furthermore, individuals with elevated emotional intelligence are adept at managing work-related stressors and challenging situations by employing positive emotion-focused strategies that mitigate the impact of negative emotions on personal happiness (Sánchez Álvarez et al., 2016). Additionally, individuals possessing high levels of emotional intelligence can establish effective interpersonal relationships through proficient communication skills within various contexts such as colleagueship dynamics or familial interactions (Uchenna and Nkechi, 2020). Simultaneously, robust interpersonal relationships and social support serve as crucial determinants of wellbeing. Individuals with elevated levels of emotional intelligence possess the ability to employ effective strategies for regulating their emotions, striking a balance between positive and negative affective states, fostering an optimistic and positive mindset, and nurturing a closely linked positive emotional state conducive to happiness. Moreover, individuals with heightened emotional intelligence exhibit sustained self-motivation, channeling their emotions in a constructive and productive manner that enhances team collaboration, work performance, job satisfaction, and a sense of accomplishment (Ouyang et al., 2015). Satisfaction and achievement represent pivotal dimensions for assessing overall wellbeing.

### 6.2 Emotional intelligence and work-family support

Based on the findings of this study, there is a significant positive relationship between the emotional intelligence of college teachers and their work-family support. Higher levels of emotional intelligence among college teachers facilitate obtaining support from work and family domains. Despite the lack of direct research on the relationship between emotional intelligence and work-family support, existing studies indicate that emotional intelligence facilitates the acquisition of social support (Zhang et al., 2022). Work-family support is considered a specific form of social support; it can be inferred that emotional intelligence may positively influence work-family support. In line with current research, emotional intelligence has been shown to reduce work-family conflict (Wittmer Jenell et al., 2023) and promote work-family balance (Pan et al., 2022). These fundamentals are closely related to work-family support. He et al. (2020) discovered that individuals with high emotional intelligence demonstrate better evaluation skills regarding their working environment, are more inclined to select organizations conducive to their personal growth, actively seek support from these organizations, and possess heightened sensitivity toward receiving social support from family and friends. Li et al. (2016) extended the conversation. They explained that emotional intelligence is closely associated with interpersonal relationships, as individuals with elevated emotional intelligence tend to exhibit proficient interpersonal skills. Individuals with high emotional intelligence are adept at maintaining harmonious relationships with colleagues, leaders, and service recipients, increasing their likelihood of receiving support from these individuals. Moreover, individuals with high emotional intelligence demonstrate proficiency in pleasing family members and fostering harmonious familial relationships, resulting in an increased likelihood of obtaining both emotional and instrumental support. For instance, when faced with difficulties, family members are more inclined to offer encouragement and proactively share household responsibilities. In summary, possessing high emotional intelligence enables individuals to enhance levels of work-family support and effectively navigate the challenges that arise between work and family domains. However, it remains unclear whether emotional intelligence is a cause or a consequence of work-family support. Some scholars argue that a family support network enhances individuals’ ability to recognize, understand, and regulate their emotions (Castañeda et al., 2023), while organizational support contributes to the improvement of emotional intelligence (Gao et al., 2024).

### 6.3 Work-family support and wellbeing

According to the findings of this study, there is a significant positive association between work-family support and the overall wellbeing (including life wellbeing, work wellbeing, and psychological wellbeing) of college teachers. In other words, an increase in the level of work-family support received by college teachers is linked to a stronger sense of wellbeing. Although no studies have directly examined the relationship between work-family support and wellbeing, existing research on work-family dynamics provides evidence that work-family support influences wellbeing. For instance, workplace support can help mitigate work-family conflict (Wangxi and Andrew, 2025), while broader social support can alleviate job burnout (Fiorilli et al., 2019). There are three primary factors contributing to this phenomenon. Firstly, work-family support can facilitate individuals in achieving a better equilibrium between their work and family responsibilities. For instance, the provision of flexible working hours enables individuals to actively engage in familial matters, thereby reducing conflicts arising from the interplay between work and family domains and mitigating their adverse consequences (Jiang and Yang, 2020). Secondly, work-family support serves as a buffer against the exacerbation of work-related stress caused by job demands (Liang, 2020). When employees confront excessive pressure and expectations at work, they are prone to experiencing fatigue and dissatisfaction. Thirdly, the provision of work-family support can enhance employees’ sense of affiliation. Organizations may offer employee coaching and training, compassionate leadership, and emotional support to foster a greater sense of belonging among employees. Simultaneously, when workers perceive organizational support, they are more motivated and engaged in their duties while also experiencing higher levels of job satisfaction. Finally, work-family support can enhance overall quality of life by facilitating a better balance between work and family responsibilities. This enables employees to effectively care for their families, engage in social activities and personal hobbies, and ultimately experience increased happiness and satisfaction in life. In essence, work-family support fosters individuals’ equilibrium between work and family commitments, leading to heightened life satisfaction and positive psychological wellbeing (Chen et al., 2014).

### 6.4 The mediating effect of work-family support

According to the findings of this study, the emotional intelligence of college teachers indirectly influences their life wellbeing, work wellbeing, and psychological wellbeing through work-family support. Previous studies have demonstrated that the indirect impact of emotional intelligence on wellbeing occurs via two primary pathways: first, the stress coping pathway, wherein individuals’ emotional intelligence influences their wellbeing through effective stress management strategies (Schutte et al., 2006); second, the social capital pathway, through which emotional intelligence enhances wellbeing by improving relationship quality and organizational support (Joseph Dana and Newman, 2010). These findings are consistent with research indicating that work-family balance mediates the relationship between emotional intelligence and job satisfaction (Hemade et al., 2025). Simultaneously, the resource conservation theory supports the notion that individuals tend to utilize existing resources to acquire new ones, thereby providing theoretical backing for college teachers to employ high emotional intelligence in obtaining resources within both their work and family. Employees with elevated levels of emotional intelligence demonstrate enhanced effectiveness in utilizing work-family support resources and effectively managing the interplay between work and family responsibilities, ultimately leading to an augmented sense of wellbeing. However, the provision of work-family support also contributes significantly to the development of emotional intelligence. When organizations effectively implement measures to support employees in managing their work and family responsibilities, it facilitates the regulation and balance of these demands, mitigates negative emotions, and promotes psychological wellbeing. Such supportive measures may encompass flexible work arrangements, family leave policies, assistance with childcare or parental care, among others. The implementation of these supportive measures can effectively alleviate employees’ work-related stress, facilitate their emotional regulation, and enhance their ability to achieve a harmonious integration between work and family responsibilities. Consequently, emotional intelligence can serve as a mediating variable that amplifies the positive impact of organizational support on job satisfaction (Suárez-Albanchez et al., 2022). Therefore, further research is warranted to ascertain the causal relationship between emotional intelligence and work-family support.

### 6.5 Theoretical implications

Compared with previous studies, this research, grounded in resource conservation theory and job demand-resource theory, elucidates the mechanism through which college teachers leverage their emotional intelligence to secure work-family support, thereby enhancing their wellbeing. Understanding the mechanism can better motivate individuals to harness their subjective initiative in pursuing wellbeing. As a mediating variable, work-family support constructs also provide a new perspective for studying the impact of work-family relationship on employee wellbeing. Using an integrated employee wellbeing construct, rather than subjective wellbeing or a single dimension of wellbeing, would also help to assess individual wellbeing across the board.

### 6.6 Practical implications

According to the findings of this study, emotional intelligence and work-family support have been identified as factors that can enhance individuals’ satisfaction with both their personal and professional lives, as well as promote positive psychological experiences. In order to foster the wellbeing of teachers in colleges, two approaches can be adopted: enhancing teachers’ emotional intelligence and providing them with additional resources and support, and colleges can implement the following specific strategies: First, enhancing emotional intelligence through structured training programs is essential. Colleges can establish regular workshops that focus on emotional recognition, regulation, and conflict resolution. These workshops should incorporate practical exercises and role-playing scenarios to ensure teachers can apply these skills in real-life situations. Additionally, creating peer support networks will allow teachers to share experiences and strategies, fostering a collaborative environment that promotes emotional growth. Second, colleges must create a positive work atmosphere by implementing open forums where faculty can express concerns and provide suggestions. This not only promotes transparency but also builds trust within the institution. Establishing accessible mental health resources, such as counseling services and support hotlines, is crucial for helping teachers manage stress effectively. Moreover, colleges should focus on providing essential resources and support by developing transparent evaluation mechanisms that outline clear criteria for assessments and promotions. Engaging faculty in the development of these criteria ensures fairness and accountability. Implementing flexible work arrangements, such as remote work options and adjustable hours, will help teachers balance their professional responsibilities with personal commitments. Finally, teachers should be encouraged to actively manage their family dynamics, and colleges can assist by offering family support programs that provide resources on improving relationships and managing work-life balance. By implementing these strategies, Colleges can significantly enhance faculty emotional intelligence and overall wellbeing, leading to improved job satisfaction and a more positive educational environment. Future research should explore the long-term impacts of these initiatives and the specific challenges institutions may encounter during implementation.

### 6.7 Limitations and future research

Although this study has enriched the research on emotional intelligence, work-family relationships, and employee wellbeing, it has several limitations. First, the reliance on cross-sectional data and design constrains our ability to infer causality between the variables under investigation. Second, while the data were collected via a contemporaneous questionnaire survey and passed the common method bias test, the potential for common method bias remains, as it cannot be entirely ruled out. Finally, the sample is exclusively drawn from Chinese colleges, lacking representation from other countries, which limits the cross-cultural generalizability of the findings. Given these limitations, future research could be enhanced in the following ways: First, by using longitudinal data to better establish causal relationships among variables. Second, through multi-source or multi-wave data collection methods to mitigate common method bias. Third, by incorporating samples from diverse cultural contexts to examine the universality of the findings. Lastly, numerous factors influence employee satisfaction, and future research should consider incorporating additional variables such as culture and situational factors of organization to better elucidate the contextual applicability of the findings from this study.

## Data Availability

The raw data supporting the conclusions of this article will be made available by the authors without undue reservation.
